# Supplementary material for: Being Stung Once or Twice by Bees (Apis mellifera L.) Slightly Disturbed the Serum Metabolome of SD Rats to a Similar Extent
Source: Int J Mol Sci. 2024 Jun 8;25(12):6365. doi: 10.3390/ijms25126365 (PMC11203678; doi:10.3390/ijms25126365)
Supplement: Supplementary file 1 [file ijms-25-06365-s001.zip › ijms-2992134-supplementary.pdf]

Supplementary Materials

Table S1. Result from Compound Name Mapping.

	HMDB	Match	PubChem	KEGG
1	HMDB01882	Dihydroxyacetone	670	C00184
2	HMDB00008	2-Hydroxybutyric acid	11266	C05984
3	HMDB00729	Alpha-Hydroxyisobutyric acid	11671	
4	HMDB00005	2-Ketobutyric acid	58	C00109
5	HMDB00208	Oxoglutaric acid	51	C00026
6	HMDB00695	Ketoleucine	70	C00233
7	HMDB00357	3-Hydroxybutyric acid	441	C01089
8	HMDB00491	3-Methyl-2-oxovaleric acid	47	C03465
9	HMDB00017	4-Pyridoxic acid	6723	C00847
10	HMDB00042	Acetic acid	176	C00033
11	HMDB00060	Acetoacetic acid	96	C00164
12	HMDB01659	Acetone	180	C00207
13	HMDB00161	L-Alanine	5950	C00041
14	HMDB00517	L-Arginine	6322	C00062
15	HMDB00044	Ascorbic acid	54670067	C00072
16	HMDB00191	L-Aspartic acid	5960	C00049
17	HMDB00043	Betaine	247	C00719
18	HMDB00062	L-Carnitine	2724480	C15025
19	HMDB00097	Choline	305	C00114
20	HMDB00094	Citric acid	311	C00158
21	HMDB00064	Creatine	586	C00300
22	HMDB01511	Phosphocreatine	587	C02305
23	HMDB00562	Creatinine	588	C00791
24	HMDB04983	Dimethyl sulfone	6213	C11142
25	HMDB00087	Dimethylamine	674	C00543
26	HMDB00108	Ethanol	702	C00469
27	HMDB00142	Formic acid	284	C00058
28	HMDB00134	Fumaric acid	444972	C00122
29	HMDB00122	D-Glucose	5793	C00031
30	HMDB03339	D-Glutamic acid	23327	C00217
31	HMDB00641	L-Glutamine	5961	C00064
32	HMDB00123	Glycine	750	C00037
33	HMDB00115	Glycolic acid	757	C00160
34	HMDB00128	Guanidoacetic acid	763	C00581
35	HMDB01873	Isobutyric acid	6590	C02632
36	HMDB00172	L-Isoleucine	6306	C00407
37	HMDB00190	L-Lactic acid	107689	C00186
38	HMDB00687	L-Leucine	6106	C00123
39	HMDB00182	L-Lysine	5962	C00047

Cont. Table S1.

40	HMDB00744	Malic acid	525	C00711
41	HMDB00691	Malonic acid	867	C00383
42	HMDB00169	D-Mannose	18950	C00159
43	HMDB01875	Methanol	887	C00132
44	HMDB00696	L-Methionine	6137	C00073
45	HMDB00201	L-Acetylcarnitine	1	C02571
46	HMDB01565	Phosphorylcholine	1014	C00588
47	HMDB00159	L-Phenylalanine	6140	C00079
48	HMDB00162	L-Proline	145742	C00148
49	HMDB01881	Propylene glycol	1030	C00583
50	HMDB00243	Pyruvic acid	1060	C00022
51	HMDB00271	Sarcosine	1088	C00213
52	HMDB00254	Succinic acid	1110	C00042
53	HMDB00251	Taurine	1123	C00245
54	HMDB00167	L-Threonine	6288	C00188
55	HMDB00906	Trimethylamine	1146	C00565
56	HMDB00925	Trimethylamine N-oxide	1145	C01104
57	HMDB00158	L-Tyrosine	6057	C00082
58	HMDB00300	Uracil	1174	C00106
59	HMDB00294	Urea	1176	C00086
60	HMDB00883	L-Valine	6287	C00183
61	HMDB00211	Myoinositol		C00137
62	HMDB00086	Glycerophosphocholine	71920	C00670
63	HMDB00725	4-Hydroxyproline	5810	C01157
64	HMDB00479	3-Methylhistidine	64969	C01152

Table S1 shows the conversion results. MSEA has a built-in tool to convert between HMDB ID and compound common names, identifiers used in PubChem and KEGG.
